# Cetuximab plus gemcitabine/oxaliplatin (GEMOXCET) in first-line metastatic pancreatic cancer: a multicentre phase II study

**DOI:** 10.1038/sj.bjc.6604983

**Published:** 2009-03-17

**Authors:** F Kullmann, S Hollerbach, M M Dollinger, J Harder, M Fuchs, H Messmann, J Trojan, E Gäbele, A Hinke, C Hollerbach, E Endlicher

**Affiliations:** 1 Department of Internal Medicine I, University of Regensburg, Regensburg, Germany; 2 Department of Internal Medicine, Academic Teaching Hospital, Celle, Germany; 3 Department of Internal Medicine I, University of Halle, Halle, Germany; 4 Department of Medicine II, University Medical Center, University of Freiburg, Freiburg, Germany; 5 Department of Gastroenterology, Hepatology and Gastrointestinal Oncology, Academic Teaching Hospital, München-Bogenhausen, München, Germany; 6 Department of Medicine III, Academic Teaching Hospital, Augsburg, Germany; 7 Department of Medicine I, University of Frankfurt, Frankfurt, Germany; 8 WISP Research Institute, Langenfeld, Germany

**Keywords:** pancreatic cancer, chemotherapy, cetuximab, gemcitabine, oxaliplatin

## Abstract

Targeting the epidermal growth factor receptor pathway in pancreatic cancer seems to be an attractive therapeutic approach. This study assessed the efficacy of cetuximab plus the combination of gemcitabine/oxaliplatin in metastatic pancreatic cancer. Eligible subjects had histological or cytological diagnosis of metastatic pancreatic adenocarcinoma. The primary end point was response according to RECIST. Patients received cetuximab 400 mg m^−2^ at first infusion followed by weekly 250 mg m^−2^ combined with gemcitabine 1000 mg m^−2^ as a 100 min infusion on day 1 and oxaliplatin 100 mg m^−2^ as a 2-h infusion on day 2 every 2 weeks. Between January 2005 and August 2006, a total of 64 patients (22 women (34%), 42 men (66%); median age 64 years (range 31–78)) were enrolled at seven study centres. On October 2007, a total of 17 patients were alive. Sixty-two patients were evaluable for baseline and 61 for assessment of response to treatment in an intention-to-treat analysis. Six patients had an incomplete drug combination within the first cycle of the treatment plan (*n*=4 hypersensitivity reactions to the first cetuximab infusion, *n*=2 refused to continue therapy). Reported grade 3/4 toxicities (% of patients) were leukopaenia 15%, anaemia 8%, thrombocytopaenia 10%, diarrhoea 7%, nausea 18%, infection 18% and allergy 7%. Cetuximab-attributable skin reactions occurred as follows: grade 0: 20%, grade 1: 41%, grade 2: 30% and grade 3: 10%. The intention-to-treat analysis of 61 evaluable patients showed an overall response rate of 33%, including 1 (2%) complete and 19 (31%) partial remissions. There were 31% patients with stable and 36% with progressive disease or discontinuation of the therapy before re-staging. The presence of a grade 2 or higher skin rash was associated with a higher likelihood of achieving objective response. Median time to progression was 118 days, with a median overall survival of 213 days. A clinical benefit response was noted in 24 of the evaluable 61 patients (39%). The addition of cetuximab to the combination of gemcitabine and oxaliplatin is well tolerated but does not increase response or survival in patients with metastatic pancreatic cancer.

Pancreatic cancer is the second leading neoplasia of the gastrointestinal tract. Only 4% of patients with adenocarcinoma of the pancreas will be alive 5 years after diagnosis (Jemal *et al*, 2004). Until now, weekly gemcitabine is accepted as the standard palliative chemotherapy for advanced pancreatic cancer, with median survival of 6 months but associated with a clinical benefit for example, improvement of pain, weight and performance status (Burris *et al*, 1997).

Louvet *et al* (2002) showed that gemcitabine combined with oxaliplatin was well tolerated and resulted in a promising response rate (30.2% for metastatic and 31% for locally advanced disease). Median progression-free survival (PFS) and overall survival (OS) were 5.3 and 9.2 months, respectively.

With respect to molecular biology, the epidermal growth factor receptor (EGFR) has shown to play an important role in the carcinogenesis of pancreatic cancer (Yamanaka *et al*, 1993; Fjällskog *et al*, 2003). Therefore, targeting the EGFR pathway seems to be an attractive therapeutic approach. In advanced pancreatic cancer, Xiong *et al* (2004) showed that cetuximab in combination with gemcitabine resulted in an improvement in response (12.2% partial response (PR), 63.4% stable disease (SD)), with a median time to progression of 3.8 months and a median survival of 7.1 months.

On the basis of these data, we assessed the activity of the combination of gemcitabine with oxaliplatin plus cetuximab in patients with advanced pancreatic cancer. As outcome of patients with locally advanced and metastatic pancreatic cancer is different (Louvet *et al*, 2002) and most trials combined both groups of patients so far, only patients with metastatic disease were included. The objectives of the trial were to determine the response rate (according to RECIST criteria), time to progression, survival, clinical benefit response and safety profile.

## Materials and methods

### Patients

Patients with histologically or cytologically proven metastatic (non-regional lymph nodes or distant metastasis) adenocarcinoma of the pancreas (stage IVb), who had received no previous chemotherapy, were included in this study. Evidence of EGFR expression was not necessary for eligibility.

Other eligibility criteria included Karnofsky performance scale ⩾70%, minimum age of 18 years, at least 6 months since the completion of any adjuvant therapy, at least 4 weeks since the completion of any radiation therapy (measurable tumour mass has to be outside the radiation field) and adequate organ function, as indicated by a white blood cell count of ⩾3000/*μ*l, haemoglobin level of ⩾9 g dl^−1^, platelet count of ⩾100 000/*μ*l, alkaline phosphatase level and serum transaminase level of ⩽5 times the upper limit of normal (ULN), total bilirubin level of ⩽2 times ULN and creatinine level of ⩽1.5 mg dl^−1^. Before treatment, all patients provided written informed consent according to each institutional standard. The treatment protocol was approved by local ethics committees.

### Treatment

Patients received cetuximab at an initial dose of 400 mg m^−2^ followed by weekly doses of 250 mg m^−2^. Patients were then observed for 30 min for signs of anaphylaxis or other infusion-related reactions (IRRs). If a patient had an IRR, the infusion time was doubled from standard time, and this increase was maintained for subsequent infusions. If a patient had a grade-3 skin toxicity, the subsequent dose of cetuximab was delayed for up to 2 consecutive weeks, with no change in dose level. If toxicity resolved to grade 2 or less within 2 weeks, treatment resumed. If a patient had a second or third occurrence of a grade-3 skin toxicity, cetuximab was again delayed for up to 2 weeks, with dose decreases to 200 and 150 mg m^−2^, respectively.

Sixty minutes after the cetuximab dose, gemcitabine was administered at a dose of 1000 mg m^−2^ over a 100 min infusion on day 1 and oxaliplatin at a dose of 100 mg m^−2^ as a 2-h infusion on day 2 every 2 weeks.

Dose modifications of gemcitabine and oxaliplatin were based on absolute neutrophil counts (ANCs) and platelet counts. Doses were decreased by 25% if the ANC nadir was between 500 × 10^6^ cells per l and 999 × 10^6^ cells per l or the platelet count nadir was between 50 × 10^6^ cells per l and 99 × 10^6^ cells per l, and drugs were withheld if the ANC nadir was less than 500 × 10^6^ cells per l or the platelet count nadir less than 50 × 10^6^ cells per l. Missed doses of gemcitabine and oxaliplatin were not administered and both drugs were restarted when the platelet count had risen to 100 × 10^6^ cells per l or above and ANC to 1000 × 10^6^ cells per l or more. Gemcitabine and oxaliplatin were not withheld if the cetuximab infusion was suspended because of skin toxicity.

During the entire treatment antiemetics (anti-5HT_3_, steroids) were given. Furthermore, patients received full supportive care.

### Study evaluations

Evaluations before and during the treatment consisted of a complete medical history and physical examination, assessment of Karnofsky performance status and laboratory studies, including haematological and biochemical profiles, computed tomography or magnetic resonance imaging of the abdomen and chest or other body areas with disease involvement. Imaging studies were performed before every fourth cycle and at follow-up visit (unless the patient discontinued for disease progression) to assess tumour response.

Clinical benefit was evaluated according to the definition of Rothenberg *et al* (1996).

Patients were followed up until death.

### Response criteria and toxicity

Tumour response was evaluated and graded using RECIST criteria (Therasse *et al*, 2000). Toxicity was categorised using the National Cancer Institute Common Toxicity Criteria (version 2.0). All patients who received the study treatment were included in the analysis of toxicity in an intention-to-treat analysis.

### Statistical design

The primary end point was objective response (OR), defined as the proportion of patients whose best response was either PR or complete response (CR) in the intent-to-treat population. Secondary end points included disease control rate (defined as the proportion of patients whose best response was CR, PR or SD), PFS and OS. OS was defined as the time from the beginning of chemotherapy to death. PFS was defined as the time from the beginning of chemotherapy to disease progression or death, whichever occurred first. The event-related end points were estimated by the Kaplan–Meier method. An exact version of the *χ*^2^ test for trend was applied to assess the association between skin rash and response.

The sample size was calculated according to a two-stage optimal design by Simon (1989). On the basis of the findings by Louvet *et al* (2002) on the gemcitabine/oxaliplatin combination, an observed OR of less than 20% was considered as futile, whereas, in contrast, the experimental combination regimen would be regarded as a very promising candidate for further evaluation, if an OR of 40% could be achieved. This resulted in a total sample size of 54 evaluable patients, with an interim analysis after 19 patients, allowing to stop futility. Statistical analysis was performed using the SPlus software (Insightful Corp., Seattle, WA, USA).

## Results

### Enrollment and patient characteristics

Between January 2005 and September 2006, a total of 64 patients were enrolled in the study.

Eighteen patients were enrolled at the co-ordinator site of Regensburg, 14 in Celle, 8 in Halle, 7 in Freiburg, 6 in Augsburg, 6 in Munich (Bogenhausen) and 5 in Frankfurt.

Baseline characteristics of the evaluable patient population (*n*=62) are shown in Table 1.

**Figure 1:**
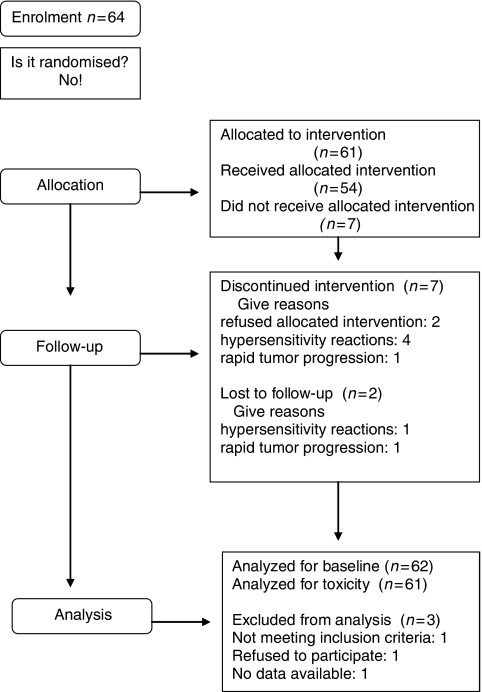
Flowchart of patients.

Of the 64 patients, 61 were available for assessment for response to treatment in an intention-to-treat analysis; one patient refused to take part in the study after giving consent for participation. One patient could not be treated within the study due to a rapid deterioration of performance status, and in one patient, no documentation was available. The study flowchart is shown in Figure 1.

Six of evaluable patients had an incomplete drug combination within the first cycle of the treatment plan (*n*=4 hypersensitivity reactions to the first cetuximab infusion, *n*=2 refused to continue therapy).

A total of 499 cycles of chemotherapy were administered in the study. Patients received a median number of seven cycles (range 1–43). Treatment delays occurred in 27% of all cycles, mainly (47%) due to organisational reasons (patients’ wish) and only in 12% because of haematological toxicity. In 21%, chemotherapy doses had to be reduced mainly due to peripheral polyneuropathy (39%) and haematological toxicity (9%).

### Toxicity

The combination of cetuximab with gemcitabine and oxaliplatin was generally well tolerated. Reported grade 3/4 toxicities (% of patients) were leukopaenia 15%, anaemia 8%, thrombocytopaenia 10%, diarrhoea 7%, nausea 18%, infection 18% and allergy 7%. Cetuximab-attributable skin reactions occurred as follows: grade 0: 20%, grade 1: 41%, grade 2: 30% and grade 3: 10%. Clinically relevant toxicity is summarised in Table 2.

### Efficacy

**Figure 2:**
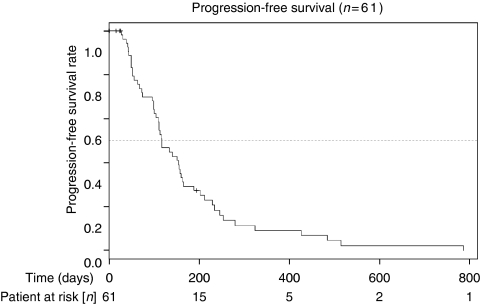
Kaplan–Meier plots for progression-free survival.

**Figure 3:**
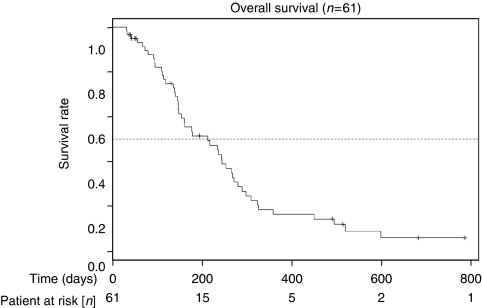
Kaplan–Meier plots for overall survival.

Overall median follow-up time was 154 days (range 15–786). Table 3 shows responses. One patient (2%) had a CR and 19 patients (31%) a partial remission, with an overall response rate of 33%. There were 31% patients with stable and 36% with progressive disease or missing restaging due to early dropout. Median time to progression was 118 days, with a median OS of 213 days. Figures 2 and 3 show the OS and PFS curves. Forty-seven patients died. A clinical benefit response was noted in 24 out of 61 patients (39%).

Table 4 shows a rash *versus* response analysis. The severity of skin rash was associated with a higher likelihood of achieving tumour response (*P*=0.0031).

In all, 29 out of 61 (47%) patients received second-line therapy, mainly a fluorouracil-based chemotherapy.

## Discussion

Until now, gemcitabine has been widely accepted as a standard treatment in patients with advanced pancreatic cancer although numerous trials evaluated a variety of combination protocols with two and even three drug regimens (Adler *et al*, 2007).

In a phase II study of Louvet *et al* (2002), gemcitabine combined with oxaliplatin resulted in a high response rate (31%), and median OS was also promising for patients with metastatic and locally advanced disease with 8.7 and 11.5 months, respectively. The results of a following phase III trial confirmed the efficacy and safety of gemcitabine combined with oxaliplatin but failed to show a statistically significant advantage in terms of OS compared with gemcitabine (Louvet *et al*, 2005). Nevertheless, analogous to the phase II trial, median survival times were identical in both arms for locally advanced patients (30% of total population), whereas for metastatic patients (70% of the total population), the median survival time was 6.7 months in the gemcitabine arm and 8.5 months in the gemcitabine/oxaliplatin arm.

Recently, data of a pooled analysis of Heinemann *et al* (2007) showed that in patients with good performance status, the combination of gemcitabine with a platinum analogue, such as oxaliplatin or cisplatin, significantly improves PFS and OS as compared with single-agent gemcitabine in advanced pancreatic cancer.

Therefore, considering the promising results of biological agents (Xiong *et al*, 2004), we thought that the addition of cetuximab to the combination of gemcitabine and oxaliplatin would be consequently the next step for evaluation in patients with advanced pancreatic cancer. Furthermore, this is the first study to our knowledge including only patients with metastatic pancreatic carcinoma for the evaluation of the activity of a palliative first-line platin-based chemotherapy protocol.

In our study on 64 patients with metastatic pancreatic carcinoma, the addition of cetuximab to the combination of gemcitabine and oxaliplatin was well tolerated and exhibited a response rate of 33%. The median time to PFS was 3.9 months and OS was 7.1 months. These findings are not superior to the results achieved in the earlier studies of gemcitabine and oxaliplatin alone. Meanwhile, Cascinu *et al* (2008) reported data of a phase II trial in which patients with advanced pancreatic cancer were randomly assigned to treatment with gemcitabine and cisplatin alone *versus* gemcitabine and cisplatin plus cetuximab. In all, 61 out of 84 (73%) patients had metastatic disease. Seven out of 40 (17.5%) patients had an OR rate in the cetuximab group and 5 out of 41 (12.2%) in the non-cetuximab arm. No significant differences between the groups were noted in the median PFS or in the median OS. Median PFS was 3.4 months in the cetuximab group and 4.2 months in the non-cetuximab group. Median OS was 7.5 months and 7.8 months, respectively. Interestingly, toxic effects were not increased by cetuximab, and at least 33 out of 61 (54%) patients with metastatic disease received a second-line fluorouracil-based chemotherapy. The authors concluded from their data that cetuximab does not add any valuable activity to a combination of gemcitabine and cisplatin. The findings of Cascinu *et al* (2008) are in agreement with those of a phase III study evaluating cetuximab in combination with gemcitabine compared with gemcitabine alone in advanced pancreatic cancer, published only in the abstract form so far (Philip *et al*, 2007). Seven hundred and thirty-five patients were randomly enrolled in this latter trial and 78% had metastatic disease. The median survival was 6 months in the gemcitabine arm and 6.5 months in the gemcitabine plus cetuximab arm, failing to show a clinically significant advantage of the addition of cetuximab to gemcitabine.

Interestingly, consistent with the data of Xiong *et al* (2004), our results indicate a possible correlation between response and severe acne rash. Acne rash may therefore play a role as a surrogate marker of the efficacy of EGFR inhibition.

In conclusion, the addition of cetuximab to a combination of gemcitabine and oxaliplatin does not result in a prolonged survival in comparison with earlier studies evaluating gemcitabine and oxaliplatin alone. Molecular analyses to identify genetic alterations in pancreatic cancer with a therapeutic potential are warranted for the future.

## Figures and Tables

**Table 1:** Baseline characteristics

**Characteristic**	
Patients (*n)*	62
Age, years (median, range)	64.5 (31–78)
Male/female	41/21
	
*Performance status (*n)	
90–100	33 (63%)
80	13 (25%)
70	6 (12%)
	
*Distant metastasis (*n)	
Lymph nodes (abdominal/pelvic)	8 (13%)
Liver	58 (93%)
Lung	8 (13%)
Other	22 (35%)
Adjuvant treatment (before start of trial), *n*	1 (2%)

**Table 2:** Treatment-related grade3/4 toxicities

**Toxicity**	**NCI grade 3**	**NCI grade 4**
Anaemia	2 (3%)	3 (5%)
Leukopaenia	9 (15%)	—
Thrombocytopaenia	6 (10%)	—
Nausea	11 (18%)	—
Vomiting	4 (7%)	1 (2%)
Diarrhoea	4 (7%)	—
Infection	7 (11%)	4 (7%)
Allergy	3 (5%)	1 (2%)
Skin toxicity	6 (10%)	—

**Table 3:** Response to treatment (intention to treat)

	***N*=61**
Complete response, *n* (%)	1 (2%)
Partial response, *n* (%)	19 (31%)
Stable disease, *n* (%)	19 (31%)
Progressive disease, *n* (%)	10 (16%)
No restaging/early dropout, *n* (%)	12 (20%)
Objective response, *n* (%)	20 (33%)
Disease control, *n* (%)	39 (64%)

**Table 4:** Rash and response to treatment

	**Rash grade 0**	**Rash grade 1**	**Rash grade ⩾2**
*N*=61	12 (20%)	25 (41%)	24 (39%)
Complete response, *n* (%)	—	—	1 (**4%**)
Partial response, *n* (%)	1 (8%)	6 (24%)	12 (**50%**)
Stable disease, *n* (%)	1 (8%)	12 (48%)	6 (**25%**)
Progressive disease/ No restaging/early drop out, *n* (%)	10 (83%)	7 (28%)	5 (**21%**)

The bold values signify objective response (CR HR) by rash grade. *P*=0.0031.
